# Metagenomic characterization of swine slurry in a North American swine farm operation

**DOI:** 10.1038/s41598-021-95804-y

**Published:** 2021-08-20

**Authors:** Akshaya Ramesh, Emily S. Bailey, Vida Ahyong, Charles Langelier, Maira Phelps, Norma Neff, Rene Sit, Cristina Tato, Joseph L. DeRisi, Annette G. Greer, Gregory C. Gray

**Affiliations:** 1 Weill Institute for Neurosciences, University of California, San Francisco, CA 94158 USA; 2 Department of Neurology, University of California, San Francisco, CA 94158 USA; 3 Division of Infectious Diseases, Duke University School of Medicine, Durham, NC USA; 4 Julia Jones Matthews Department of Public Health, Texas Tech University Health Sciences Center, Abilene, TX USA; 5 Chan Zuckerberg Biohub, San Francisco, CA 94158 USA; 6 Division of Infectious Diseases, University of California San Francisco, San Francisco, CA 94158 USA; 7 Department of Biochemistry and Biophysics, University of California, San Francisco, CA 94158 USA; 8 Department of Bioethics and Interdisciplinary Studies, Brody School of Medicine, North Carolina Agromedicine Institute, East Carolina University, Greenville, NC USA; 9 Duke Global Health Institute, Duke University, Durham, NC USA; 10 Emerging Infectious Disease Program, Duke-NUS Medical School, Singapore, Singapore; 11 Global Health Center, Duke Kunshan University, Kunshan, China

**Keywords:** Diseases, Molecular medicine, Environmental sciences

## Abstract

Modern day large-scale, high-density farming environments are inherently susceptible to viral outbreaks, inadvertently creating conditions that favor increased pathogen transmission and potential zoonotic spread. Metagenomic sequencing has proven to be a useful tool for characterizing the microbial burden in both people, livestock, and environmental samples. International efforts have been successful at characterizing pathogens in commercial farming environments, especially swine farms, however it is unclear whether the full extent of microbial agents have been adequately captured or is representative of farms elsewhere. To augment international efforts we performed metagenomic next-generation sequencing on nine swine slurry and three environmental samples from a United States of America (U.S.A.) farm operation, characterized the microbial composition of slurry, and identified novel viruses. We assembled a remarkable total of 1792 viral genomes, of which 554 were novel/divergent. We assembled 1637 *Picobirnavirus* genome segments, of which 538 are novel. In addition, we discovered 10 new viruses belonging to a novel taxon: porcine *Statoviruses*; which have only been previously reported in human, macaques, mouse, and cows. We assembled 3 divergent *Posaviruses* and 3 swine *Picornaviruses*. In addition to viruses described, we found other eukaryotic genera such as *Entamoeba* and *Blastocystis*, and bacterial genera such as *Listeria*, *Treponema*, *Peptoclostridium* and *Bordetella* in the slurry. Of these, two species *Entamoeba histolytica* and *Listeria monocytogenes* known to cause human disease were detected. Further, antimicrobial resistance genes such as tetracycline and MLS (macrolide, lincosamide, streptogramin) were also identified. Metagenomic surveillance in swine fecal slurry has great potential for novel and antimicrobial resistant pathogen detection.

## Introduction

In modern large-scale farming environments, humans and dynamic populations of livestock are often in frequent and prolonged close contact. Swine are recognized to harbor multiple pathogens which may spillover to humans^1, 2^. In such farms, viruses are readily transmitted within the farm and across species. The transmission occurs through direct contact and environmental pathways such as via aerosol, feces, and water^3–7^. Globalization of the swine industry has contributed to the emergence and global spread of pathogens of swine: porcine epidemic diarrhea virus spread from China to the United States of America (U.S.A.) in 2013, affecting 50% of the U.S.A. breeding herds; and African swine fever that emerged in 2007 and re-introduced in China in 2018 which resulted in the culling of over 300 million pigs and direct economic losses of $141 billion U.S.A. dollars^8, 9^. Of particular concern is the movement of zoonotic pathogens between livestock and farm workers and the threat of epidemic pathogen transmission to neighboring communities, exemplified by the H1N1 “swine flu” pandemic in 2009 that originated from influenza A viruses circulating in pig populations^1, 2, 10–13^. These examples highlight the need to build a complete unbiased global picture of swine pathogens to understand patterns of emergence and spread^13^. Unbiased metagenomic sequencing is an important and proven tool to preemptively investigate the potential disease-causing microbial burden of livestock environments^14–16^.

A 2018 review article, analyzing over 57,000 publications over the last 50 years, has identified the top 40 priority pathogens (including zoonotic) for swine diseases, with viruses (40%) and bacteria (37.5%) accounting for a majority of the pathogens^9^. Swine farms have been the subject of prior metagenomic sequencing efforts, mostly outside North America^17–21^. These investigations have yielded a plethora of rich information, especially with respect to possible viral pathogens, and suggest that additional exploratory screening should take place^17–21^.

While obvious, engaging agribusinesses in emerging or reemerging pathogens surveillance is challenging^22^. Major industry objections that must be overcome include the biosecurity risks of permitting researchers to enter farms, the harm that specimen collection may cause the animals, and the often unspoken concerns that surveillance may reveal occupational hazards that could damage revenue^23^. Some of these objections can be met using noninvasive metagenomic surveillance techniques^24, 25^.

In this study, we build upon previous efforts using metagenomic sequencing methods to examine swine fecal slurry samples from a U.S.A. farm for molecular evidence of pathogens. Our primary goal was to determine if indirect and noninvasive swine slurry sampling could yield robust microbial detections in order to support pathogen surveillance at the human-animal interface in North America. We focus our results on the viral families identified, given that they account for the highest number of pathogens in the priority swine (and zoonotic) pathogen list, and global economic impact^8, 13, 26^. Here, we report that even limited sampling can reveal a rich, highly informative pathogen landscape, replete with both known and novel viral entities.

## Results

### Overview of mNGS results

Nine fecal slurry samples were sequenced at an average depth of 71,585,514 reads (Interquartile Range (IQR): 53,008,460–94,664,576), with an average of 22,402,963 non-host reads (IQR: 16,368,802–26,021,652). Three environmental samples were sequenced at an average depth of 36,654,247 reads (IQR: 8,048,069–53,255,240), with an average of 12,818,139 non-host reads (IQR: 1,986,958–18,978,324). The water control was sequenced at a depth of 920,074 reads.

**Figure 1:**
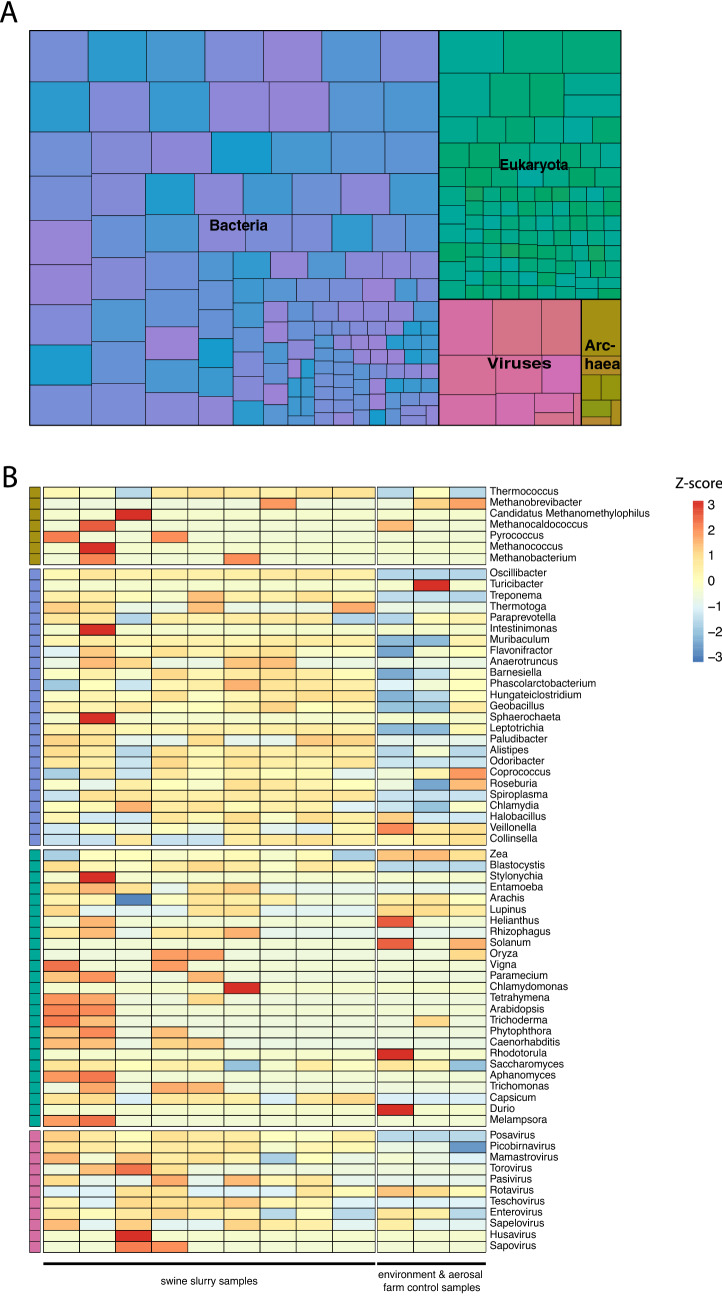
Microbial composition of swine slurry: (**A**) Treemap of microbial kingdoms (genus count) identified across all the nine swine slurry samples. (**B**) Top 25 most abundant genera (combined NT + NR reads per million) within each kingdom identified in the slurry samples.

A total of 270 microbial genera meeting our criteria were found in the slurry samples. Figure 1A shows a breakdown of the microbial composition of the pig slurry. Bacteria were the most prevalent kingdom (54.81%), followed by Eukaryota (38.52%), Viruses (4.04%) and Archaea (2.59%). Figure 1B represents the top 25 most abundant genera within each kingdom that were identified. The most prevalent bacteria genera were *Oscilibacter* and *Treponema*. Among viruses, *Posavirus*, *Picobirnavirus* and *Mamastrovirus* were the most prevalent genera. As mentioned in the introduction, we have focused our results mainly on the viruses identified in the swine slurries, and offer a brief description of the microbial compositions of other kingdoms. Supplemental Table 1 has detailed breakdown of all the microbial genera found across all nine slurry and 3 farm environment (environment and aerosol) samples.

**Table 1:** Antimicrobial resistance genes detected in 9 slurry samples and 3 environmental samples.

AMR gene	# Gene Alleles detected in samples (% prevalence in samples)	Mean rpM
Tet	67 (100%)	80
MLS	66 (100%)	82
AGly	29 (92%)	79
Bla	17 (100%)	78
Phe	3 (25%)	88
Nim	2 (17%)	102
Sul	1 (8%)	66

*AMR* antimicrobial resistance, *rpM* reads per million mapped reads, *tet* tetracycline, *MLS* macrolide, lincosamide, streptogramin, *AGly* aminoglycoside, *Bla* beta-lactamas, *Phe* phenicol, *Nim* nitromidazole, *Sul* sulfa.

### Bacteria and antimicrobial resistance genes

The most prevalent bacterial genera present across all samples (nine slurry + three environment) are: *Veillonella* (predominantly *V. parvula*), *Roseburia* (predominantly *R. hominis* and *R. intestinalis*), *Phascolarctobacterium* (predominantly *P. faecium*), *Hungateiclostridium* (predominantly *H. clariflavum* and *H. thermocellum*), *Flavonifractor* (predominantly *F. plautii*), *Coprococcus* (predominantly *C. catus*), *Barnesiella* (predominantly *B. viscericola*), *Listeria* (predominantly *L. monocytogenes*), *Chlamydia* (predominantly *C. suis*), *Mycoplamsa* (predominantly *M. fermentans* and *M. capricolum*), and *Treponema* (predominantly *T. brennaborense*) (Fig. 1B, Supplemental Table 1). In addition to these, other notable bacterial genera identified included *Peptoclostridium* (predominantly *P. acidaminophilum*) and *Bordetella* (predominantly *B. bronchialis*) found in 75% and 50% of the samples, respectively.

In addition to bacteria, we detected a diversity of antimicrobial resistance genes.

The most prevalent class of antibiotic resistant genes detected in the nine slurry (and 3 environmental) samples were tetracycline and MLS (macrolide, lincosamide, streptogramin) both of which were detected in 100% of the samples using a 10% allele coverage level (Table 1).

### Eukaryotes

Eukaryotic genera found in these swine slurry samples (nine slurry + three environment) included *Trichomonas* (predominantly *T. vaginalis*) (25%), *Entamoeba* (predominantly *E. histolytica)* (41.66%), and *Blastocystis* (predominantly *B. hominis)* (75%) (Supplemental Table 1). Additionally, plant genera such as *Lupinus*, *Capsicum*, *Zea, Saccharomyces* and *Arachis* were also present in over 50% of the samples.

### Viruses

#### Picobirnavirus

*Picobirnavirus* was the most prevalent genus in the slurry samples (Fig. 1B). A total of 638 RNA-dependent RNA polymerase (RdRP) segments and 1033 capsid/ORF segments greater than 1 KB were assembled across all nine slurry samples; no viral genomes could be assembled from the farm environment samples (viral reads to *Picobirnavirus* were identified in two of three environment samples). Phylogenetic analysis of all complete RdRP segments in this study (354/638) and all complete *Picobornavirus* genomes from NCBI indicates that this genus was highly diverse, and belong to genogroups I and II. The porcine picobirnaviruses were not limited by host range (Supplemental Fig. 1). BLASTN and BLASTX percent identities of all RdRP segments had closest GenBank relatives with percent identities between 70.96 and 100% (mean: 85.93%, 50 segments had no BLASTN hits) and 48.1–99.56% (mean: 84.07%, 3 segments had no BLASTX hits), respectively. Of these, 31/683 RdRP genomes were divergent, and less than 75% identical (lower identity between BLASTN and BLASTX) to their closest sequence relative on GenBank. Capsid/ORF gene segments, had BLASTN and BLASTX percent identities of 63.4–100% (mean: 76.4%, 17 segments had no BLASTN hits) and 19.26–93.1% (mean: 42.2%, 5 segments had no BLASTX hits), respectively. 507/1033 capsid/ORF segments were divergent.

#### Posavirus

**Figure 2:**
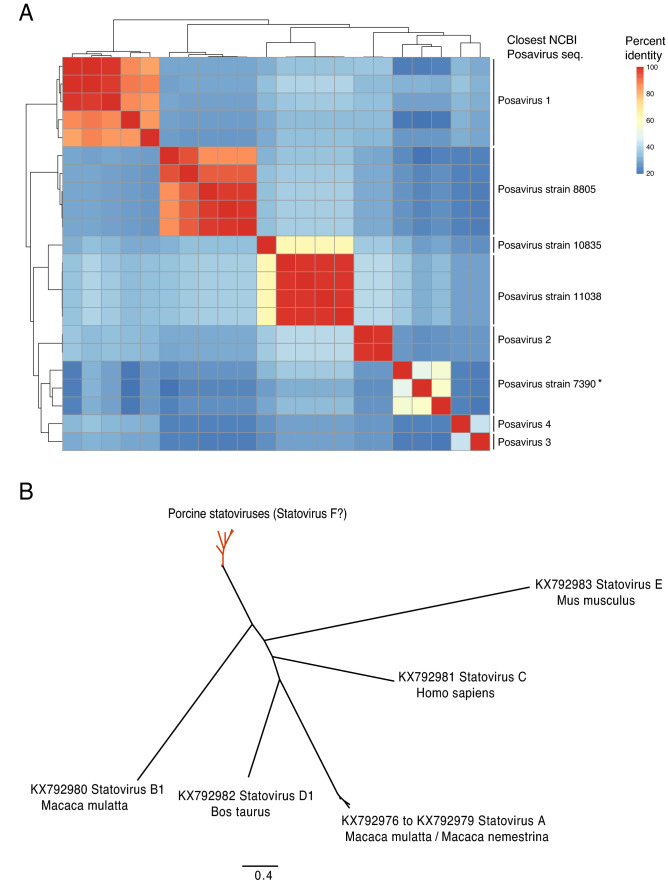
(**A**) Similarity matrix of *Posavirus* assembled in this study. (**B**) Maximum Likelihood phylogenetic tree of a novel taxon of porcine *Statovirus* genomes assembled in this study.

Followed by *Picobirnavirus*, *Posavirus* was most prevalent in the slurry samples (Fig. 1B, Supplemental Table 1). A total of 22 genomes, grouped into 10 clusters (Fig. 2A), were assembled across the nine samples. No reads to *Posavirus* were detected across farm environment samples. Among these, 17 genomes had > 85% identities (BLASTN, BLASTX) compared to existing *Posavirus* genomes on GenBank: *Posavirus* 1 (5/17), *Posavirus* strain 8805 (5/17), *Posavirus* strain 11,038 (4/17), *Posavirus* 3 (1/17), *Posavirus* 4 (1/17) and *Posavirus* strain 10,835 (1/17). Of the remaining five genomes, 2/22 were most closely related to *Posavirus* 2 (2/22) with BLASTN and BLASTX of ~ 74% and ~ 78% respectively. The other 3/22 genomes, were highly divergent, with no BLASTN hits, and closest BLASTX hits were ~ 50% [mean nucleotide % identity: 50.2% (range: 50.15–50.39%); mean amino acid % identity: 50.2% (range: 39.89–39.96% 40.07%)] to their closest relative on GenBank (KT833072.1). These 3 divergent genomes had pairwise identities of 49.46, 57.99 and 55.05% similar to each other, and a mean of 26.57% (IQR: 23.07–30.58%) to the other 19 *Posavirus* genomes at the nucleotide level.

#### Porcine astrovirus

A total of 36 genomes, of Porcine astrovirus types 1, 2, 4 and 5 were assembled across the nine samples. While reads to porcine astrovirus were identified in all three farm environment samples, no genomes could be assembled. BLASTN and BLASTX of these viral genomes to the NCBI server revealed that the percent identities of their respective closest genomes to GenBank had a mean of 90.4% and 90.04%, with a range between 88.56–92.49% and 63–96.9%, respectively.

#### Sapelovirus, Enterovirus G, Teschovirus A, Swine picornavirus and Pasivirus A

Forty-nine genomes across four different genera in the Family *Picornaviridae* were identified in the nine samples. Specifically, eight genomes of Enterovirus G representing five different enterovirus types were present. Interestingly, only three of eight genomes also had a papain-like cysteine protease sequence (PL-CP) in the 2C–3A junction region. Thirteen genomes of *Teschovirus* A were identified across all nine samples, representing eight different types. Four *Sapelovirus* A genomes, all consisting of a single serotype were assembled in four different samples. Four genomes of swine picornavirus (% identities between genomes: 58.54–99.9% (median: 58.79%)), were assembled across three different samples. Three of the four swine *picornavirus* genomes, were less than 75% identical at the NT level, [VP1 (mean: 61.74, range: 57.38–70.32%), 2C: (mean: 68.23, range: 67.93–68.85%), 3C: (mean: 60.92, range: 58.63–65.33%), 3D: (mean: 67.38, range: 67.14–67.86%)] compared to their closest swine picornavirus genomes in GenBank (LC113907), classifying them as a novel species. Of these three novel species, 2 were almost identical (pairwise identity 99.27%) to each other. Finally, 19 *Pasivirus* A genomes across five samples were identified, with % identities between genomes 70.55–99.6% (median: 75.45%). No divergent viruses were detected in the other viral families: *Sapelovirus*, *Enterovirus* G, *Teschovirus* A, and *Pasivirus* A. While no genomes were assembled from the farm environmental samples, reads to these genus of viruses were identified in the control environment samples as well (see Supplemental Table 1 for further breakdown).

#### Statovirus

Ten porcine *Statoviruses*, a novel taxon described previously only in human, macaques, mouse, and cows, was identified across five slurry samples (Fig. 2B). Of these, 5/10 were > 99% similar to each other at the NT level and 2/10 were 90% similar to each other at the NT level, with the other 3/10 genomes sharing between 75 and 85% percent identity.

#### Other viral genera

Additionally, other viral genera of interest that were identified in the pig slurry samples included *Sapovirus, Torovirus, Husavirus, Rotavirus*. Of these, there was sufficient sequencing coverage to assemble one *Sapovirus* and three *Torovirus* genomes successfully.

Finally, a large number of reads (63,043.25–158,443.0213 NT rPM) were labelled as “uncategorized”, (i.e. taxa with neither family nor genus classification were found in all samples), including in the water control (23,680.98 rPM). Due to our exclusion criteria, microbial genera identified in the water control were not reported in the samples. It is likely that further analyses of these “uncategorized” reads, will be revealing, and identify further previously unidentified and uncharacterized microbes.

## Discussion

Modern high-density swine farming practices facilitate novel pathogen emergence and horizontal transmission, and increase the risk of zoonotic spillover to other species, including humans^27^. This is due to multiple factors including crowded livestock conditions, the frequent introduction of immunologically naïve young animals, the frequent movement of animals^28^, and environmental transmission pathways. As most high-density animal operations process animal waste in large lagoons, these pools pose an additional transmission risk to neighboring farm and communities through ground water seepage and large rainwater runoffs. GPS mapping of animal production locations in North Carolina can demonstrate that the proximity of animal facilities can pose public health risks of viral spread from one farm to another through simple measures such as feed delivery^29–31^. Communication between farmers regarding biosecurity and emerging viruses is essential to the public health of animals and humans working in production environments as well as surrounding communities.

Our pilot metagenomic study of swine slurry captures the virome, both known and novel, as well as microbes across the tree of life, revealing tremendous diversity and complexity. Given that viruses account for the largest number of swine pathogens, and their global economic impact, we have focused the results of our study on this kingdom^8, 13^. In this study alone, we report 554 novel viruses of unknown pathogenicity and zoonotic potential. Further, *Entamoeba histolytica* and *Listeria monocytogenes*, known human pathogens of concern were identified. We also demonstrated how fecal slurry can be used to examine antimicrobial resistance genes in bacteria prevalent in swine.

In this study, *Picobirnaviruses* represented a substantial fraction (30%, 1671 genomes) of the viral sequences. *Picobirnaviruses* are non-enveloped double-stranded RNA viruses, typically consisting of two segments and are often found in the fecal matter of a wide range of species, sometimes associated with diarrhea^32^. A large number of diverse *Picorbirnaviruses* (n = 1236) have also been previously reported in camel feces^33^. This study adds 1671 genomes, to the existing 2540 genomes of *Picobirnaviruses* currently publicly available in NCBI, greatly increasing our sequence knowledge of this group of viruses within the Durnavirales order. The true host of *Picobirnaviruses*, whether it be eukaryotic (including fungal), or prokaryotic, remains unresolved^34^. Whether *Picobirnaviruses* are causes, or indirect markers of enteric disease also remains controversial.

*Posaviruses* (Porcine stool-associated RNA viruses) are highly diverse members of *Picornavirales* and widely observed and first discovered in fecal samples from pigs^35–37^. In addition to previously known *Posavirus* types, we identified three highly divergent *Posaviruses*. Further, we report the first porcine *Statovirus* (Stool Associated Tombus-like virus); to-date these viruses have only been reported in the gastrointestinal tract of four other mammals: humans, macaques, cows and mice^38^. As is the case with *Picobirnaviruses,* the true host and pathogenicity of these viruses are unclear.

Unlike the above mentioned viral genera, there is evidence of porcine host infection by Astroviruses, another positive-sense single stranded non-enveloped virus that has been studied extensively^35, 39, 40^. Aside from enteric and respiratory infection, porcine astrovirus type 3 in particular has been associated with encephalomyelitis in pigs^41^. However in this study, we only detected porcine astrovirus types 1, 2, 4 and 5.

In addition to viral families discussed above, other microbial species that are known to be pathogenic to humans were detected. *E. histolytica*, a human pathogen associated with intestinal and extraintestinal infections, responsible for 5 million infections annually, was identified in 41.66% of the samples^42^. Further, *Listeria monocytogenes*, a serious foodborne illness with a high mortality and hospitalization rates was identified in all slurry samples studied^43^. We also detected antimicrobial resistance genes in these samples. The genes identified have been implicated in both human disease and food safety^27, 44^; however due to a lack of a understanding of how genomic detection of these genes compares to the gold-standard, the significance of these findings are unclear^45^.

Our exploratory pilot study has important limitations. First, only a small number (nine) of slurry samples were collected from two farms. Second, these samples were collected over a limited time period of 6 months; larger and wider sampling is likely to reveal temporal dynamics of the microbial species. Finally, we have focused our analyses and discussion only on viral families and further analyses of the other microbial families (including “uncategorized” reads) is likely to yield a more complex and diverse microbial portrait. Nonetheless, from two moderately-sized farms with an average of 3800 head of swine, we identified a rich and diverse microbial landscape, including 554 novel viruses using our small sample set. More extensive and longitudinal studies of fecal slurry from a large US farm (20,000 head) or a megafarm in China (> 84,000 head)^46^ would likely yield even greater diversity. Just as studies of human sewage have assisted public health officials in understanding polio virus^47^ and SARS-CoV-2 transmission^48^, our results suggest that periodic assessments of swine farm fecal slurry might be an effective noninvasive approach to novel pathogen surveillance at industrialized swine farms. Such surveillance should be attractive to the swine industry as an early warning method for swine pathogen incursions^49^ and additionally, assist public health officials in assessing possible swine zoonoses public health threats^50^.

## Materials and methods

### Site enrollment

During the fall of 2018, A North Carolina, USA, swine farm with two geographic locations was identified to participate in this preliminary study. Each barn had 12 pens with a center hallway and a fully slatted concrete floor over a deep pit to hold feces, urine, and waste water.at Location #1 and slightly larger at location #2. This farm held on average 3800 head of swine during a year. Pits were emptied up to three times per year and recharged with recycled water. Farm personnel collected up to two (6 oz. syringes) slurry samples per week from the pit of each production location.

This study was granted exemption from review status by the Institutional Animal Care and Use Committee at Duke University on the grounds that the research did not include direct sample collection from animals.

### Sample collection and processing

Slurry is defined as the feces and urine from pigs and the waste water used to remove the urine and feces from the pig pens^51^. Nine slurry samples from two swine barns containing finishing pigs were collected from approximately 5–10 cm below the surface of pits. Three environmental samples (two aerosol and one surface swab) were also collected and were frozen at − 20 °C until shipped to our laboratory (a maximum of 24 h). Aerosol sample and environmental swabs were collected as previously described^52^. Frozen samples and completed surveys were transported overnight to the Duke One Health Research Laboratory. Dates and pre-assigned sample numbers were used for sample tracking.

Slurry samples were diluted by methods previously described^25^ and genomic DNA was extracted using the Zymo Research *Quick*-DNA Miniprep Kit (Cat. No. D3024). Extracted samples were shipped to the Chan-Zuckerberg Biohub (San Francisco, California) and stored at − 80 °C until processed by molecular methods.

### Library preparation and metagenomic next-generation sequencing (mNGS)

RNA was extracted and Libraries were prepared using NEBNext Ultra II RNA Library Prep Kit for Illumina (Cat. No. E7770) using Mayday et al. (2019)^53^.

External RNA Controls 103 Consortium (ERCC) [ThermoFisher, catalog no 4456740] collection spike-in controls were used in all samples. Libraries were pooled and sequenced on a NextSeq to generate 150 base pair, paired-end sequences.

### Microbe identification and bioinformatic analysis

Microbial pathogens were identified from raw sequencing reads using IDseqV3.9, a cloud-based, open-source bioinformatics platform recently described in Ramesh et al. (2019), Saha et al. (2019), and Kalantar et al. (2020)^54–56^. Only microbial genera present at over 10 rpM, both at the nucleotide (NT) and protein (NR) levels were reported. Additionally, to control for any potential background contaminants, all microbial genera identified in the water control were excluded.

To more comprehensively characterize the genomes of viruses in the metagenomic dataset, genomes of identified microbes were assembled de novo using St. Petersburg genome assembler (SPAdes)^57^ and annotated using Geneious v10.3.2. Contigs assembled that were 50% or greater than the Reference genome were included in the downstream analysis. For the *Picobirnavirus* genus, contigs greater than 1 KB were included in the analysis. For a given viral species, the assembled genomes were then aligned using the default settings in MUSCLEv3.8.1551. ModelTest-NGv0.1.5 was used to identify the best-fitting evolutionary model for each viral species. We reconstructed a maximum-likelihood phylogeny using RAxML-ngv0.6.0 using default settings (bootstrap = 200).

### Criteria for identification of novel or divergent virus

Picobirnavirus virus was categorized as novel/divergent if they were less than 75% identical to their closest relative on GenBank (BLASTN and BLASTX)^58^. *Posavirus* were classified as divergent if their BLASTN and BLASTX identities were below 50%^35^. Novel species in swine picornavirus was identified based on ICTV guidelines^59^.

### Antimicrobial resistance (AMR) assessment

To identify antimicrobial resistance genes present in the metagenomic dataset, SRST2 was used^60^. AMR genes with at least 10% allele coverage were considered positive.

### Ethics approval

This study was granted exemption from review status by the Institutional Animal Care and Use Committee at Duke University on the grounds that the research did not include direct sample collection from animals.

## Supplementary Information

Supplementary Figure 1.

Supplementary Figure Legend.

Supplementary Table 1.

## Data Availability

All of the raw data generated for this study is available PRJNA683083. Assembled genomes can be found at MW977024—MW977661, MW977662—MW978694, and MW504477-MW504597.
