# Supplementary material for: Metagenomic characterization of swine slurry in a North American swine farm operation
Source: Sci Rep. 2021 Aug 20;11:16994. doi: 10.1038/s41598-021-95804-y (PMC8379149; doi:10.1038/s41598-021-95804-y)
Supplement: Supplementary file 1 — Supplementary Figure 1. [file 41598_2021_95804_MOESM1_ESM.pdf]

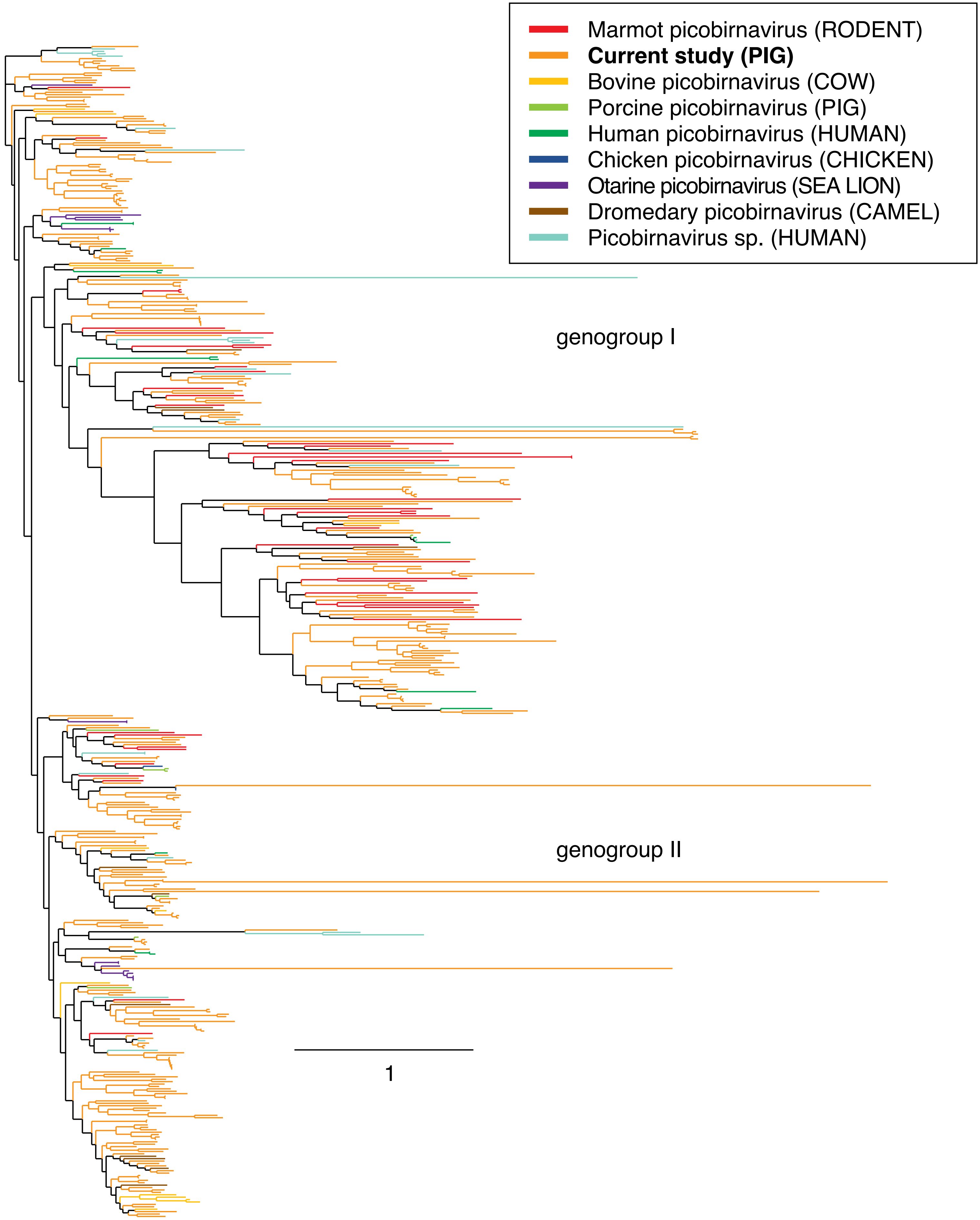

Marmot picobirnavirus (RODENT)
Current study (PIG)
Bovine picobirnavirus (COW)
Porcine picobirnavirus (PIG)
Human picobirnavirus (HUMAN)
Chicken picobirnavirus (CHICKEN)
Otarine picobirnavirus (SEA LION)
Dromedary picobirnavirus (CAMEL)
Picobirnavirus sp. (HUMAN)
genogroup I
genogroup II
1
