# Supplementary material for: Metagenomic characterization of swine slurry in a North American swine farm operation
Source: Sci Rep. 2021 Aug 20;11:16994. doi: 10.1038/s41598-021-95804-y (PMC8379149; doi:10.1038/s41598-021-95804-y)
Supplement: Supplementary file 2 — Supplementary Figure Legend. [file 41598_2021_95804_MOESM2_ESM.docx]

**Supplemental Figure Legend**

Figure 1. Maximum Likelihood phylogenetic tree of all complete 354 Picobirnaviruses RdRp segments assembled in this study and all complete Picobornavirus genomes downloaded from NCBI.
